# Diagnostic significance of microRNA-1255b-5p in prostate cancer patients and its effect on cancer cell function

**DOI:** 10.1080/21655979.2021.2009413

**Published:** 2021-12-11

**Authors:** Yuling Zhao, Xiaochun Tang, Yifan Zhao, Yan Yu, Shuzhen Liu

**Affiliations:** a Department of Laboratory, Traditional Chinese Medical Hospital of Huangdao District Qingdao, Qingdao Shandong, China; b Department of Blood Transfusion, Traditional Chinese Medical Hospital of Huangdao District Qingdao, Qingdao Shandong, China; c Department of Minimally Invasive Intervention Center, Qingdao Municipal Hospital, Qingdao Shandong, China; d Urology Department, Shanghai Pudong New Area People’s Hospital, Shanghai, China; e Department of Disinfection Supply Center, Traditional Chinese Medical Hospital of Huangdao District Qingdao, Qingdao Shandong, China

**Keywords:** miR-1255b-5p, prostate cancer, diagnosis, proliferation, migration, invasion

## Abstract

Discerning between indolent and aggressive types is a big challenge of prostate cancer clinically to guide the adequate therapeutic regimen. We aimed to examine the relationship between miR-1255b-p expression and prostate cancer and elucidate the function of miR-1255b-5p in prostate cancer. miR-1255b-5p were measured using Quantitative Real-Time PCR from the blood 103 benign prostate hyperplasia (BPH) and 153 prostate cancer patients (117 indolent cases and 36 upgrading cases). Using receiver operating characteristic (ROC) curve analysis, the discriminating ability of miR-1255b-5p was accessed between BPH and prostate cancer participants, or indolent and aggressive type. Using CCK-8 and Transwell assays, the function of miR-1255b-5p on prostate cancer cells was investigated. The levels of miR-1255b-5p were significantly raised in prostate cancer patients when compared with BPH participants. MiR-1255b-5p level can distinguish prostate cancer patients from BPH or indolent type from aggressive type. Downregulation of miR-1255b-5p can suppress the proliferative, invasive, and migratory capacity, but this effect can be eradicated by *EPB41L1* inhibition. The measurement of miR-1255b-5p in blood may provide a new noninvasive approach for the diagnosis of prostate cancer. miR-1255b-5p may become a potential therapeutic target for prostate cancer.

## Introduction

Though much progress has been archived, prostate cancer remains the most common malignancy assaulting men and a major cause of cancer-related mortality in men, with 375,304 new deaths in 2020 worldwide [1]. With the characteristic of profound genomic diversity and remarkable heterogeneity, prostate cancer differs from indolent to aggressive, which caused a highly variable prognosis in patients [2]. Instead of the noninvasive nature of indolent tumors, aggressive ones tend to metastasize locally or even distantly to other organs, causing poor outcomes or significant mortality [3]. Currently, the main screening methods for prostate cancer were transrectal ultrasound, digital rectal examination (DRE), or serum Prostate-Specific Antigen (PSA). However, each test can identify only a proportion of this cancer, and the risk of overdiagnosis and overtreatment is usually posed by the PSA screen, owing to its lack of specificity [4–6]. A big challenge of this tumor clinically was to discern between indolent and aggressive disease to guide the adequate therapeutic regimen [7]. To address this challenge, molecular biomarkers are highly sought for improving prostate cancer diagnosis.

microRNAs (miRNAs) are a series of noncoding single-stranded RNA molecules, conserved in evolution and short in size [8]. Within the RNA-induced silencing complex, miRNAs negatively moderate translation of targeting messenger RNAs (mRNAs) through binding to their 3ʹ untranslated region (UTR) or sometimes 5ʹ UTR [8]. Due to the complexity of crosstalk between miRNAs and mRNAs, miRNAs are involved in almost all key cellular processes as estimated, such as cell proliferation, apoptosis, differentiation, and migration [9,10]. Growing works have focused on the potential use of miRNAs as biomarkers for cancer diagnosis, including prostate cancer [11–13]. Evaluation of specific miRNAs exclusively expressed in clinical samples has been a good way to detect cancer [14]. For the reclassification of prostate cancer, miRNAs also showed strong ability in distinguishing indolent tumors from aggressive ones [15,16]. In previous literature, miR-1255b-5p presented down-expression in renal cell carcinoma and hepatocellular carcinoma. The exosomal miR-1255b-5p content in colorectal cancer impacted cell invasion, EMT-related protein expression, and telomerase stability [17–19]. In addition, the urine miR‑1255b‑5p reached excellent specificity and sensitivity in the diagnosis of invasive bladder cancer [20]. However, its diagnostic value in prostate cancer has not been thoroughly studied [21].

We, therefore, presumed that if miR-1255b-5p is dysregulated, its role in prostate cancer would be multiplex. We aimed to determine the expression level of miR-1255b-5p in serum from patients of indolent or aggressive prostate cancer, using benign prostatic hyperplasia (BPH) as control. We tried to furtherly investigate the distinguished ability of miR-1255b-5p for patients with benign prostatic hyperplasia and patients with low-risk tumor patients, indolent cases, and upgrading cases. The effect of miR-1255b-5p on cell function was also involved.

## Materials and methods

### Study population

Prostate cancer patients (n = 153) were included in the study. They were diagnosed at Shanghai Pudong New Area People’s Hospital between 2010 and 2015. In these patients, based on the biopsy histopathological result, a total of 105 patients were categorized as Gleason score 6, while 48 patients were categorized as Gleason score 7, which all consider as low-risk ones. During the following-up period, cases (n = 117) were categorized into an indolent population and upgrading population (n = 36) according to the development of the disease. Their clinical information was retrieved from medical records and list in Table 1. Another 103 patients with BPH were enrolled as control. All study participants provided written informed consent for the usage of blood samples for research purposes. The study was approved by the Institutional Ethical Committee of Shanghai Pudong New Area People’s Hospital, and the written consent of the ethical committee was obtained.

**Table 1.** Clinicopathological characteristics of prostate cancer patients and association with miR-1255b-5p expression

Variables	Number (n = 153)	miR-1255b-5p expression		*P*
		Low (n = 66)	High (n = 87)	
Prostate-specific antigen (ng/mL)	median value = 5.62 (2.02–20.49)			
Age (Years)				0.792
< 63	83	35	48	
≥ 63	70	31	39	
Gleason score at diagnosis				**0.002**
6	105	54	51	
7	48	12	36	
Tumor stage				**0.016**
I	132	62	70	
II	21	4	17	
Smoking statue				
Nonsmoker	64	27	37	0.841
Smoker	89	39	50	
Family history of prostate cancer				
No	143	60	83	0.265
Yes	10	6	4	
Positive cores (n)				
<10	82	43	39	**0.13**
≥10	71	23	48	

### Whole blood collection and processing

Whole blood was collected from the participant by nurse practitioners via ulnar vein puncture before the prostate biopsy into PAXgene Blood RNA Tubes (BD, USA). Then the tubes were centrifugated for 10 minutes at 4,000 × g. After centrifugation, the supernatant was aspirated and discarded softly and completely. The pellet was resuspended with 4 mL RNase-free water, centrifuged for 10 minutes at 4,000 × g, and discarded the supernatant.

### Cell lines preparation

Six human prostate cell lines, RWPE-1 (CRL-11609), RWPE-2 (CRL-11610), 22Rv1 (CRL-2505), LNCaP (CRL – 1740), PC-3 (CRL-1435), and DU145 (HTB-81), were originally from the ATCC (USA). RWPE-1 and RWPE-2 cell lines were cultured in Keratinocyte serum-free medium (SFM, Invitrogen, USA, 17,005–042) containing bovine pituitary extract and epidermal growth factor according to the manufacturer guide. 22Rv1, LNCaP, DU145, and PC-3 cell lines were RPMI 1640 medium (Invitrogen, USA, C11875500BT) with 10% Fetal Bovine Serum (Ausbian, Australia). The culture was incubated at 37°C in an incubator filled with a 5% CO_2_ in an air atmosphere. Cells (< passage 5) at ~80% confluence were detached for passaging according to ATCC recommendation and/or further experimental use.

Transient transfection of 22Rv1 and DU 145 cells was carried out with Lipofectamine 2000 (Invitrogen, USA) as per manufacturer protocol. miR-1255b-5p mimic (miR-1255b-5p), mimic negative control (NC), miR-1255b-5p inhibitor (anti-1255b-5p), inhibitor negative control (anti-NC), *EPB41L1* siRNA (si-*EPB41L1*), and nonspecific siRNA (si-NC) were purchased from Dharmacon (Shanghai, China). Briefly, cells were plated onto 6-well plates one day before the transfection and maintained in serum-free medium. The following day, exponentially growing 22Rv1 and DU 145 cells (1 × 10^5^ cells/well) were transfected with either 20 nmol miRNA mimic, miRNA inhibitor and/or *EPB41L1* siRNA using lipofectamine 2000 (Invitrogen, Grand Island, NY) according to manufacturer instructions.

### RNA extraction and quantification

Total RNA extraction and purification was achieved using PAXgene Blood RNA Kit (Qiagen, USA) from blood samples and the RNeasy Mini Kit (Qiagen, USA) from cells according to the respective user guide. The quantification of miR-1255b-5p and *EPB41L1* mRNA was conducted by Quantitative Real-Time PCR (qRT-PCR). For quantification of miR-1255b-5p, complementary DNA (cDNA) was synthesized using Transcriptor First Strand cDNA Synthesis Kit (Roche, USA) and NCode™ VILO™ miRNA cDNA Synthesis Kit (Invitrogen, USA). For quantification of mRNA, cDNA was made using a High-Capacity cDNA Reverse Transcription Kit (Life Technologies, USA). Platinum SYBR Green qPCR SuperMix (Life Technologies, USA) and the specific primers were mixed with cDNA for each qPCR. The test was carried out at QuantStudio™ 7 Pro Real-Time PCR System (Appliedbiosysterms, USA). The primers used were listed in Table 2. The experimental parameters were set as follows: 95°C for 3 min (initial denaturation); 40 cycles of denaturation at 95°C for 5 s, annealing at 56°C for 10 s, and extension at 72°C for 25 s; and 65°C for 5 s and 95°C for 50 s (final extension). Data acquisition was carried out using the ABI Quantstudio 7pro (Applied Biosystems, USA). The obtained data were normalized to the level of U6 or GAPDH, and data were processed with the 2^−ΔΔCT^ method.

**Table 2.** Primers sequence

Primer name	Sequence 5′ → 3′
miR‐1255b‐5p	CTCAACTGGTGTCGTGGAGTCGGCAATTCAGTTGAGAACCACTT (stem‐loop primer)
	GGGCGGATGAGCAAAGA (forward)
	AACTGGTGTCGTGGAGTCGGC (reverse)
U6	CTCGCTTCGGCAGCACA (forward)
	AACGCTTCACGAATTTGCGT (reverse)
EPB41L1	AGGAAACCACGCCGAGACACAA (forward)
	GGTGGATGAGTTTGCTGTTGGG (reverse)
GAPDH	GGAGCGAGATCCCTCCAAAAT (forward)
	GGCTGTTGTCATACTTCTCATGG (reverse)

### Cell proliferation assay

Commercially purchased Cell Counting Kit-8 (Glpbio, USA) was used for the determination of cell proliferation [22]. According to the product datasheet, 48 hours post-transfection in 22Rv1 and DU 145 cells, four lines of wells in a 96-well plate were inoculated with 2 × 10^3^ cells/well of each group: anti-NC-transfected, anti-miR-1255b-5p-transfected, anti-miR-1255b-5p/si-NC-transfected, or anti-miR-1255b-5p/si-*EPB41L1*-transfected. Cell proliferation was measured on 0, 24, 48, and 72 h using Cell Counting Kit-8 (Glpbio, USA) at a spectrophotometer (Multiskan MK3, Thermo, USA) at 450 nm.

### Cell migration and invasion assays

BioCoat Matrigel Invasion Chamber and Transwell chambers (Corning, USA) were purchased for cell invasion and migration assays, respectively [23]. The assays were carried out with 22Rv1 and DU 145 cells seeded in the upper chambers which were pre-loaded with serum-free medium. And then the cells were allowed to move to the lower chambers containing medium with 10% FBS beforehand (for migration) or 15% FBS (for invasion). Inserts were then stained, and the passed cells were photographed and counted using a light microscope.

### MiRNA Target Prediction and Plasmid construction

The target prediction of miR-1255b-5p was performed with TargetScan (http://www.targetscan.org/vert_72/), miRWalk (http://mirwalk.umm.uni-heidelberg.de/) and miRDB (http://mirdb.org/) algorithms, using keyword ‘miR-1255b-5p’ or ‘microRNA-1255b-5p’. The candidature of *EPB41L1* as miR-1255b-5p specific targets was further evaluated. Annealed custom oligonucleotides containing the putative target binding sites on 3ʹ-UTR of *EPB41L1* (2904–2910 nt.) were cloned into pmiR-GLO reporter vectors (Promega) to construct wt – *EPB41L1*. To construct a mutant vector (mut-*EPB41L1*), the binding site sequences were mutated (replacing CUUU by GAAA, and CUCAUCC by GAGUAGG) of EPB4L1 was inserted into the pmiR-GLO reporter vectors.

### Luciferase assays for potential binding sites

Anti-miR-1255b-5p or miR-1255b-5p transfected 22Rv1 and DU 145 cells were transfected with pmirGLO vectors. Then the luciferase activity was tested at 48 hours after transfection at a Dual-Luciferase Reporter Assay System (Promega, Australia) as per the manufacturer’s instructions [24]. The final data was shown as relative luciferase activity by firefly luciferase/Renilla luciferase.

### Statistical analysis

The statistical analyses were carried out with SPSS software or GraphPad Prism software. Student’s t-test, one-way, or two-way Analysis of Variance was used to determine statistical comparisons between experimental groups. The chi-squared test was used for differences in miR-1255b-5p expression among different clinicopathological key data. We performed receiver operating characteristic (ROC) curve analysis to evaluate the diagnostic value of miR-1255b-5p level in prostate cancer. A *P* value of 0.05 or less was significant.

## Results

Based on the hypothesis that dysregulation of miR-1255b-5p may happen in prostate cancer, we first determined the level of miR-1255b-5p in cancerous and normal tissues or cells. As we expected, miR-1255b-5p was lowly expressed in prostate tissues and cells than those in normal ones. Interestingly, the level of miR-1255b-5p in indolent cases and upgrading cases is also obviously different. Then the diagnostic value was evaluated and the effect on cell function was accessed.

### The expression level of miR-1255b-5p

**Figure 1.**
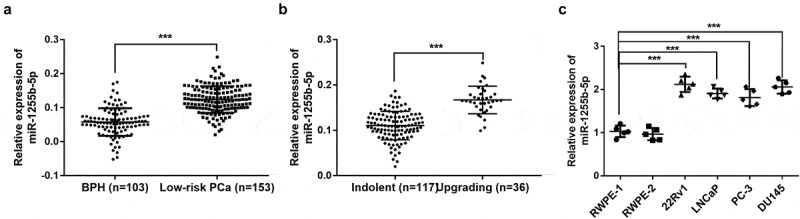
miR-1255b-5p presented an increased level in the blood samples from low-risk prostate cancer and cancer cell lines. **A**. The expression of miR-1255b-5p in blood from low-risk prostate cancer (PCa) patients and benign prostatic hyperplasia (BPH). **B**. The level of miR-1255b-5p in blood from indolent population and upgrading population. **C**. The level of miR-1255b-5p in prostate cell lines (RWPE-1, RWPE-2, 22Rv1, LNCaP, PC-3, and DU145). ***P < 0.001

From the results of qRT-PCR, a significantly increased level of miR-1255b-5p was detected from the blood sample of indolent prostate cancer patients compared with that from patients with BPH (*P* < 0. 01, Figure 1a). Among all prostate cancer patients, the upgrading ones owned elevated levels of miR-1255b-5p level compared with the indolent ones (*P* < 0.001, Figure 1b). Accordingly, a significant increase in miR-1255b-5p expression levels was observed in prostate cancer lines (22Rv1, LNCaP, PC-3, and DU 145) compared with normal prostate epithelial cells (RWPE-1 and RWPE-2) (*P* < 0. 01, Figure 1c).

### Correlation between miR-1255b-5p level and prostate cancer patient’s clinicopathological parameters

To explore whether miR-1255b-5p is correlated with parameters related to prostate cancer progression, Chi-square test was carried out. Firstly, we classified the prostate cancer patients into a low miR-1255b-5p expression group (n = 66) and high miR-1255b-5p expression group (n = 87) according to the median value of miR-1255b-5p level (0.12). From Table 1, higher miR-1255b-5p level was associated with tumor stage (*P* = 0.016) and Gleason score (*P* = 0.002). These results imply that miR-1255b-5p expression prominently correlated with prostate cancer progression.

### Diagnostic performance of serum miR-1255b-5p for prostate cancer patients

**Figure 2.**
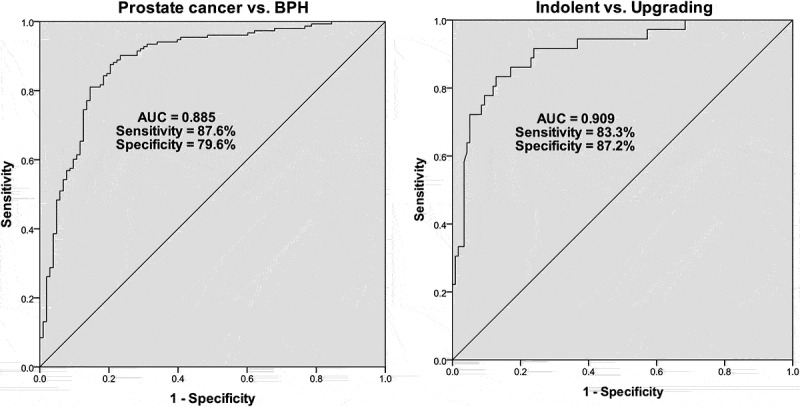
Receiver operating characteristic (ROC) curve analyses corresponds to miR-1255b-5p expression levels. **A**. ROC curve to discriminate low-risk prostate cancer patients from benign prostatic hyperplasia (BPH) individuals. **B**. ROC curve to discriminate upgrading prostate cancer from indolent ones

We next attempted to estimate the diagnostic performance of miR-1255b-5p in discriminating low-risk prostate cancer from BPH, the indolent tumor, and upgrading tumor. Based on the expression level of miR-1255b-5p, the ROC curves were plotted respectively, and the index was calculated. As shown in Figure 2a, miR-1255b-5p could well discriminate low-risk prostate cancer patients from the control (the BPH subjects) with an area under the curve (AUC) value of 0.885, and the sensitivity and specificity at cutoff value were 87.6% and 79.6%, respectively (*P* < 0.001, 95%CI: 0.841–0.929). As to the indolent tumor and upgrading tumor, miR-1255b-5p could distinguish the ones with upgrading progression from the indolent type of prostate cancer with an AUC of 0.909 (sensitivity = 83.3%, specificity = 87.2%, *P* < 0.001, 95%CI: 0.853–0.965, Figure 2b). Thus, the analysis suggested that serum miR-1255-5p had a high power in discrimination of BPH and prostate cancer, as well as the indolent type and upgrading type of prostate cancer.

### EPB41L1 3ʹUTR is directly targeted by miR-1255b-5p

**Figure 3.**
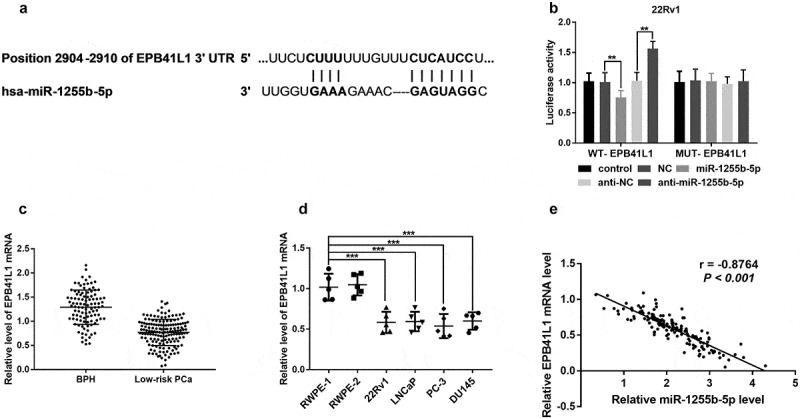
*EPB41L1* 3ʹUTR is targeted by miR-1255b-5p. **A**. The binding sites at *EPB41L1* 3ʹUTR with miR-1255b-5p. **B**. The dual-luciferase reporter assay verified the binding relationship between EPB41L1 3ʹUTR and miR-1255b-5p. **C**. The relative expression level of *EPB41L1* mRNA was reduced in blood from low-risk prostate cancer (PCa) patients compared with that from benign prostatic hyperplasia (BPH) by qRT-PCR. **D**. The relative expression level of *EPB41L1* mRNA in prostate cell lines (RWPE-1, RWPE-2, 22Rv1, LNCaP, PC-3, and DU145) by qRT-PCR. **E**. The negative correlation between the level of miR-1255b-5p and EPB41L1 mRNA expression

In this study, three bioinformatics databases were employed to predict the potential genes which target miR-1255b-5p. It was suggested that *EPB41L1* was an effective target gene of miR-1255b-5p. The expected binding sites of miR-1255b-5p and 3ʹUTR of *EPB41L1* are shown in Figure 3a. To confirm whether *EPB41L1* is the direct target of miR1255b-5p, a dual-luciferase reporter assay was performed. It was found that overexpression of miR-1255b-5p decreased the luciferase reporter activity including the wild-type sequence of the *EPB41L1*-3ʹUTR, while this influence was not found if a mutation in the target site (*P* < 0.01, Figure 3b). We also measured the *EPB41L1* mRNA expression in blood samples from prostate cancer and cells and found a significant decrease of *EPB41L1* mRNA level (*P* < 0.01, Figure 3c **and** 3d). The data of miR-1255b-5p and *EPB41L1* mRNA expression was found with a negative correlation. These findings revealed that *EPB41L1* was a downstream target gene of miR-1255b-5p.

### Effects of miR-1255b-5p on cancer cell proliferation, migration, and invasion

**Figure 4.**
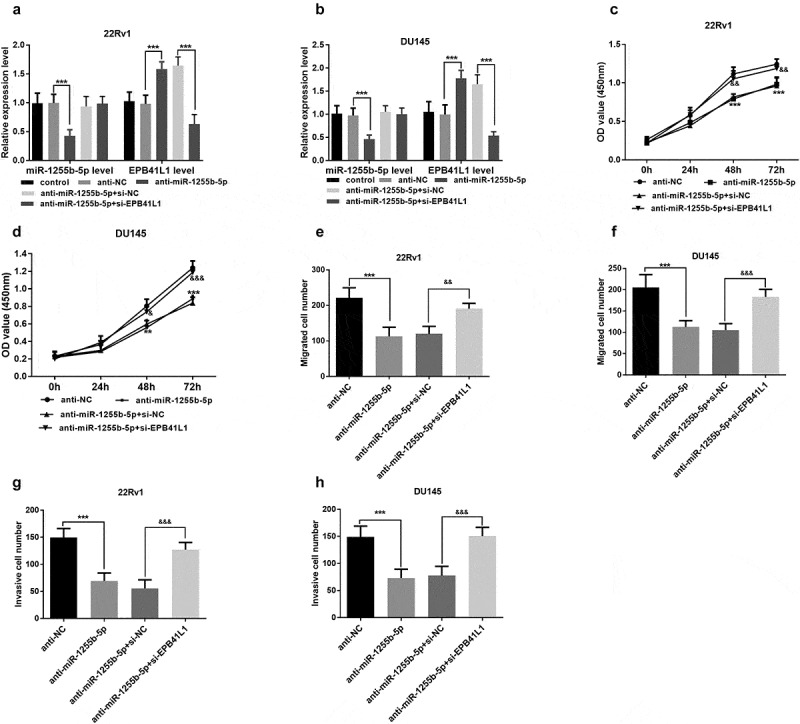
Downregulation of miR-1255b-5p inhibited the cell migration and invasion in prostate cancer cells (22Rv1 and DU145), however, these effects can be reversed by *EPB41L1* inhibition. **A** and **B**. The transfection was successful. **C** and **D**. CCK-8 assay for cell proliferation. **E** and **F**. Transwell assay for cell migration. **G** and **H** Transwell assay for cell invasion. **P < 0.01, ***P < 0.001. &*P* < 0.05, &&*P* < 0.01, &&&*P* < 0.001

Given the strong association between miR-1255b −5p and key clinicopathological parameters, the biological role of miR-1255b-5p was investigated in prostate cancer 22Rv1 and DU 145 cells. The 22Rv1 and DU 145 cells were transfected with anti-NC or anti-miR-1255b-5p single or co-transfected with si-NC or si – *EPB41L1* (*P* < 0.001, Figure 4a and 4b). To explore the *in vitro* effects of miR-1255b-5p on cell proliferation, we conducted Cell Counting Kit-8 experiments to analyze the cancer cell proliferation ability. The analysis showed that the inhibition of the miR-1255b-5p potentially decreased cell proliferation compared to the negative control (*P* < 0.001, Figure 4c and 4d). Subsequently, we assessed the cell migration and invasion using the Transwell chambers. In this regard, downregulation of miR-1255b-5p significantly suppressed the ability of prostate cancer cell migration and invasion compared to the cells transfected with negative control (*P* < 0.001, Figure 4e-4h**, Figure S1** and **S2**).

## Discussion

Active surveillance (AS) for prostate cancer is a popular option for men with Gleason 3 + 3 and low-volume 3 + 4 favorable-risk or intermediate-risk prostate cancer [25]. However, there is a wide practice variation in the selection of candidates for AS due to the uncertainty of the phenotype and misclassification of biopsy sampling grade at diagnosis or disease progression during follow-up [26]. As a result, prostate cancer patients sometimes face a difficult situation of clinical mistreatment or overtreatment [27]. Consequently, growing interest is focusing on the reclassification of prostate cancer based on molecule biomarkers [26]. The miRNAs contain characteristics that make them attractive as powerful and noninvasive diagnostic indicators of prostate cancer, such as reproducible quantification from various biologic samples, resistance to various storage conditions, and small usage of clinical samples [28,29].

In recent decades, tests based on the anomalous expression of miRNAs have been shown to improve clinical information in predicting a range of oncologic diagnoses. For instance, miRNA-222-3p was reduced in metastatic prostate cancer and might be a diagnostic biomarker. MiR‑1255b‑5p in urine can distinguish bladder cancer with 68% specificity and 85% sensitivity [20]. In this study, we evaluated whether miR-1255b-5p alterations could improve the noninvasive diagnosis or re-classification of prostate cancer patients using blood samples. For this purpose, the miR-1255b-5p expression level was tested by qRT-PCR with a result of a significant increase in cancer blood samples and cell lines. This increase was analyzed to be correlated with tumor grade and Gleason score of patients, which suggest that miR-1255b-5p expression would play a role in prostate cancer progression. MiR-1255b-5p expression also showed a differentiated expression level in upgrading tumors and the indolent ones. After assessment using ROC curves, miR-1255b-5p showed a high power in discrimination of BPH from prostate cancer, as well as the indolent type from upgrading type of prostate cancer. This study indicates that serum miR-1255b-5p is a promising biomarker for both diagnosis and reclassification of prostate cancer.

As mentioned above, to verify that miR-1255b-5p expression would influence prostate cancer progression, the cell function was determined under the condition of miR-1255b-5p downregulation. We found that miR-1255b-5p inhibition led to suppression of cell function, such as proliferation, invasion, and migration. It is reported that some individual miRNAs have pleiotropic effects either as tumor suppressors or oncogenes in different cancers, making them even more interesting and specific to cancer [30]. Commonly, an up-regulated miRNA in tumor cells is considered to be onco-miRs because it can silence the tumor suppressor genes, whereas a down-regulated one has been termed tumor suppressor which can inhibit tumor progressions, such as miR-330-3p and miR-495 [30–33]. In hepatocellular carcinoma hsa-miR-1255b presented a down-expression [18]. Zhang et al reported that the level of miR-1255b-5p was significantly lower in colorectal cancer patients compared with that in healthier and revealed the tumor-suppressing role of miR-1255b-5p in colorectal cancer [17]. Our results were in line with the previous literature about the upregulation of miR-1255b-5p in prostate cancer [21,34]. So, miR-1255b-5p may act as oncogenic miRNAs (onco-miRs) in prostate cancer.

The miRNAs can directly target mRNA and alterations in the level of cancer-related miRNAs result in the degradation of mRNA or translational repression [35]. To reveal the mechanism of miR-1255b-5p in prostate cancer progression, the downstream gene of miR-1255b-5p was presumed to be *EPB41L1* and verified then. Previously, *EPB41L1* was reported to significantly downregulate in prostate cancer tissues and be associated with biochemical recurrence [36]. Therefore, we suggest that miR-1255b-5p promotes prostate cancer cell proliferation, migration, and invasion through regulating *EPB41L1*.

## Conclusion

Taken together, we demonstrate that miR-1255b-5p is upregulated in prostate cancer blood and cell lines. In addition, miR-1255b-5p promotes prostate cancer cell proliferation, invasion, and migration *in vitro*, indicating that it may be an onco-miRNA in prostate cancer. Further, miR-1255b-5p is a powerful biomarker for prostate cancer diagnosis and reclassification for indolent or aggressive ones. These findings suggest that miR-1255b-5p may become a potential therapeutic target for prostate cancer.

## Supplementary Material

Supplemental Material

## Highlights

- miR-1255b-5p level were raised in prostate cancer patients.
- miR-1255b-5p can distinguish prostate cancer from benign prostate hyperplasia.
- miR-1255b-5p can distinguish indolent type prostate cancer from aggressive type.
- miR-1255b-5p promotes progression of prostate cancer via EPB41L1.
