# Supplementary figures and images for: Diagnostic significance of microRNA-1255b-5p in prostate cancer patients and its effect on cancer cell function
Source: Bioengineered. 2021 Dec 11;12(2):11451–60. doi: 10.1080/21655979.2021.2009413 (PMC8810192; doi:10.1080/21655979.2021.2009413)

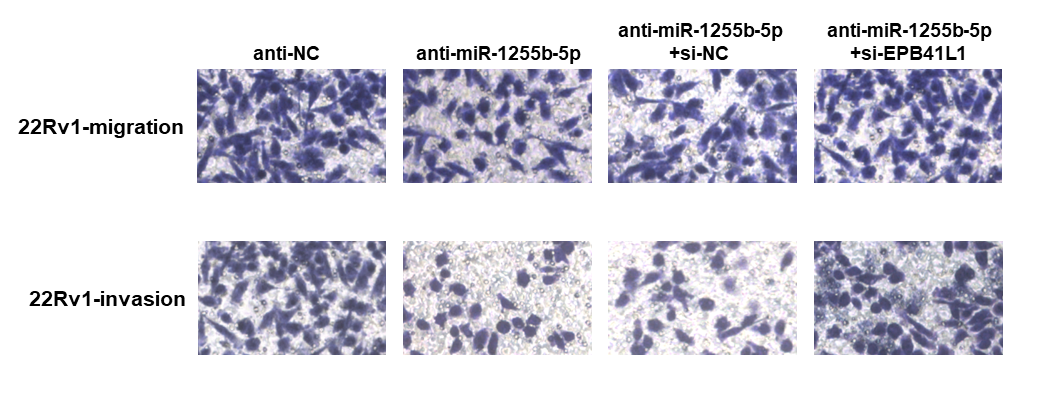

Supplement: Supplemental Material [file KBIE_A_2009413_SM7983.zip › supplementary/Figure S1.tif]

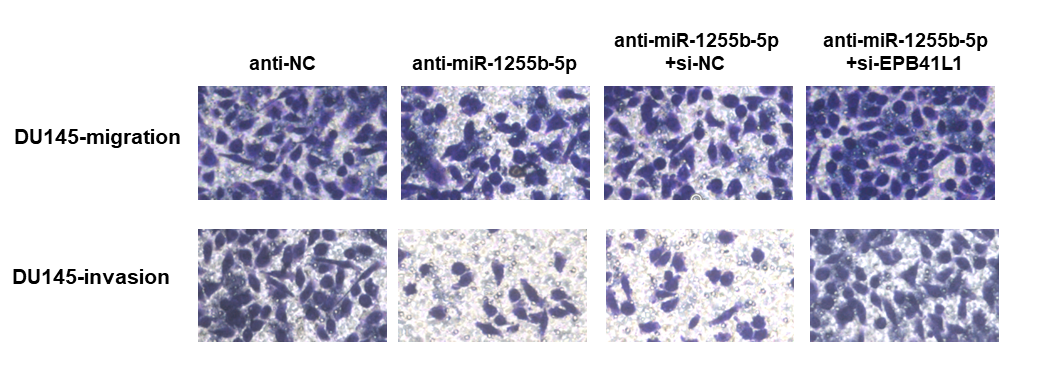

Supplement: Supplemental Material [file KBIE_A_2009413_SM7983.zip › supplementary/Figure S2.tif]
